# Robust tactile object recognition in open-set scenarios using Gaussian prototype learning

**DOI:** 10.3389/fnins.2022.1070645

**Published:** 2022-12-28

**Authors:** Wendong Zheng, Huaping Liu, Di Guo, Fuchun Sun

**Affiliations:** ^1^Department of Computer Science and Technology, Tsinghua University, Beijing, China; ^2^State Key Laboratory of Intelligent Technology and Systems, Beijing National Research Center for Information Science and Technology, Tsinghua University, Beijing, China

**Keywords:** tactile perception, object recognition, open-set recognition, Gaussian prototype learning, tactile object recognition

## Abstract

Tactile object recognition is crucial for effective grasping and manipulation. Recently, it has started to attract increasing attention in robotic applications. While there are many works on tactile object recognition and they also achieved promising performances in some applications, most of them are usually limited to closed world scenarios, where the object instances to be recognition in deployment are known and the same as that of during training. Since robots usually operate in realistic open-set scenarios, they inevitably encounter unknown objects. If automation systems falsely recognize unknown objects as one of the known classes based on the pre-trained model, it can lead to potentially catastrophic consequences. It motivates us to break the closed world assumption and to study tactile object recognition in realistic open-set conditions. Although several open-set recognition methods have been proposed, they focused on visual tasks and may not be suitable for tactile recognition. It is mainly due to that these methods do not take into account the special characteristic of tactile data in their models. To this end, we develop a novel Gaussian Prototype Learning method for robust tactile object recognition. Particularly, the proposed method converts feature distributions to probabilistic representations, and exploit uncertainty for tactile recognition in open-set scenarios. Experiments on the two tactile recognition benchmarks demonstrate the effectiveness of the proposed method on open-set tasks.

## 1. Introduction

Object recognition is a prerequisite for robotic dexterous manipulations, which is the cornerstone of many robotic applications (Li et al., 2018; Qiao et al., 2021). For example, a robot needs to know the category of an object for selecting a suitable interaction pattern or manipulation strategy during exploring the surroundings or performing manipulation (He et al., 2020; Zheng et al., 2020a). Therefore, how to effectively realize object recognition has recently attracted widespread attention in robotic research fields.

Since tactile sensing is an effective way of perceiving some physical properties of the manipulated objects through physical interaction (Luo et al., 2017), it has been extensively used in robotic tasks involving object recognition, material identification (Zheng et al., 2019), texture recognition and robotic grasp detection (Guo et al., 2021). Liu and Sun (2017) proposed a tactile recognition method for classify material identification. Xu et al. (2013) proposed a tactile identification method with Bayesian exploration. Kerr et al. (2018) used tactile data to classify the materials with the BioTAC sensor. In addition, tactile information is used as an effective complement of visual information for robotic tasks. In Liu et al. (2016), a novel visual-tactile fusion method was proposed for object recognition using joint group kernel sparse coding. Guo et al. (2017) adopted tactile information as an important complement of visual information for the robotic grasp detection task. These works have shown that tactile perception plays a significant role in robotic recognition tasks.

**Figure 1:**
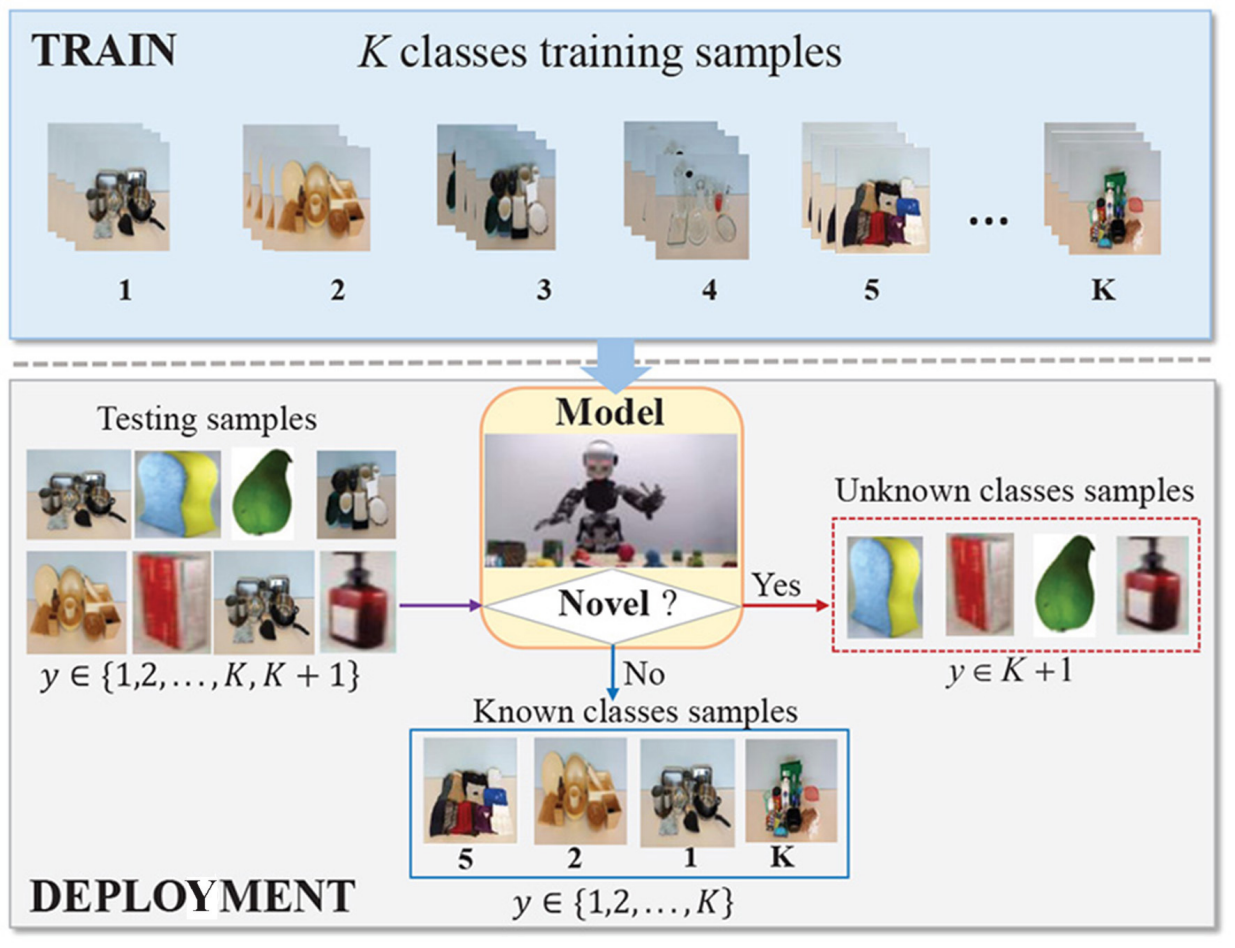
The schematic of open-set tactile object recognition. Some images are from https://sites.gatech.edu/hrl/mr-gan/.

While there are many works on tactile recognition and they have been demonstrated to be effective for some specific applications (Yi et al., 2020), they have mainly focused on constructing predictive models to classify predefined and fixed object classes in closed-set scenarios, assuming that the classes seen in testing must have appeared in training. In fact, such an assumption is usually violated in actual robotic applications (Zheng et al., 2020b). This is mainly due to that robots are commonly deployed in realistic unconstrained environments, where objects of unknown classes are regularly encountered. When observing an unknown object, these closed-set classification methods incorrectly categorize it as one of the known classes with high confidence. As classifier prediction in robotic applications can trigger some kind of costly robotic action, such misclassification can be catastrophic and is often not acceptable. Thus, it is necessary to investigate robust tactile recognition in open-set scenarios, which is also referred to open-set tactile recognition. The schematic is shown in Figure 1, where robots should have the dual ability of unknown detection and known classification.

To the best of our knowledge, tactile object recognition of open-set scenarios is still unexplored research in the robotic field. Similar to other open-set recognition, open-set tactile recognition also faces the core challenge of how to not only correctly classify samples from the known classes but also effectively detect and flag unknown examples as the novel. Many methods have been proposed to handle this problem in the literature. The mainstream methods attempt to utilize thresholding to reformulate open-set recognition as a closed-set classifier. As feature distribution of training samples is not explicitly considered in their learning objectives, the learned features generally have excessive intra-class variance. The inter-class distance can even be smaller than the intra-class distance in the learned feature space. This makes it difficult to set an appropriate threshold that well separates known from unknown.

In addition, another technical solution aims to collect unknown samples for training a (*K*+1)-class model, where *K* is the number of known categories and all unknowns are treated as an additional category. The strategy is simple and intuitive, but it usually requires large-scale training data to represent the large numbers of unknowns in open scenarios. However, collecting sufficient tactile data is difficult for training due to the complex collection process and constraints of robot-object physical interactions. Hence, constructing an effective model for open-set tactile recognition is still an open question.

As we know, humans can effectively recognize objects in open environments based on template or prototype matching. Motivated by the recognition mechanism, we propose an uncertainty estimation model for open-set tactile object recognition in this work. The framework consists of two main components, which are the feature extractor and the class prototypes. The feature extractor simulates the perception system of humans for transforming the raw sensing data into abstract representations. Moreover, the prototypes for each category serve as abstract memories of the corresponding category in the brain. By matching the tactile features (abstract representation) with prototypes (classes memories), the proposed model performs object recognition. During inference, if the feature of a test tactile sample can not match well with all the prototypes of the known classes, it will be considered as the unknown.

To this end, the learned features of each known class are characterized by a Gaussian distribution in our framework. As known samples follow the prior distributions, those test samples located in low probability regions will be recognized as unknown by the model. Meanwhile, for the test samples from known classes, the model will compute its probabilities over all known classes and classify it as the class with the highest probability. To explicitly enforce training samples following Gaussian distributions, we introduce a likelihood regularization term to the classification discriminant function during training. In addition, we further add a classification margin to make each cluster more compact and further improve the generalization of the model. The main contributions are summarized as follows:

1. In this paper, we specifically address tactile object recognition in open-set scenarios. To this end, a novel Gaussian Prototype Learning method is proposed, which is suitable for both unknown detection and known classification.
2. We introduce a likelihood regularization term to explicitly enforce training samples following Gaussian distributions. In addition, we further introduce a classification margin to make each cluster more compact, which is more beneficial for unknown detection.
3. We perform comprehensive experimental evaluations of our proposed method on publicly available tactile datasets. The experimental results demonstrate the effectiveness of the proposed method.

Please note that our proposed open-set tactile recognition is not just a matter of the robot filling in gaps in its knowledge base. Instead, we aim to enable robots will be able to continually expand the scope of the knowledge to learn new unknown classes over time in an active learning manner. That is to say, at any particular point in time the model needs to be able to detect and reject unseen data belonging to unknown tasks or classes. These unknown data could be utilized and learned with another algorithm in some human-in-the-loop system at a later stage. We believe that this research will aid in active learning and continual learning in open-set conditions, which can serve as the first step toward building lifelong robot tactile recognition systems.

In the following, related works are briefly reviewed in Section 2. In Section 3, we describe the problem of tactile open-set object recognition. Section 4 details the framework architecture and learning model of the proposed method. The experimental results and analysis are given in Section 5.

## 2. Related work

In this section, the main related works are briefly reviewed from two aspects: *tactile object recognition* and *open-Set Recognition*.

### 2.1. Tactile object recognition

Object recognition is a fundamental perceptual capability for many robot applications (Meyer et al., 2020). While vision enables robots to have excellent visual recognition capabilities (Deng et al., 2019; Han et al., 2019), it is not always effective for object recognition in practical tasks (Yang et al., 2019). This is mainly due to that objects of similar appearance can have very different physical properties, which may not be easily obtained visually (Deng et al., 2022). Tactile sensing is an important perception modality, of which the interactive nature allows it to convey rich and diverse tactile information, such as texture, roughness, or stiffness (Li et al., 2020). It is crucial for robots to explore and learn the mechanical properties of manipulated objects, especially when interacting with unknown objects in practical environments.

Considering its effectiveness in perceiving environments, tactile information has been extensively adopted in a variety of robot recognition tasks. Liu and Sun (2017) proposed a tactile material recognition model with semantic labels, which improved the identification performance. Kerr et al. (2018) utilized BioTAC sensor to collect tactile data, and then these data are used to classify the materials. Yuan et al. (2018) used GelSight tactile sensor to recognize 11 properties of the clothes, which aim to help the robot understand their material properties. Based on a hybrid touch approach, Taunyazov et al. (2019) developed an effective tactile identification framework for texture classification. More recently, Gu et al. (2020) proposed an event-based tactile object recognition method with a spiking graph neural network using electronic skins.

Although the mentioned tactile-based recognition methods have been successfully applied in some specific robotic tasks, most of them are deployed under a closed-set condition. Such a closed-set scenario is practically unfeasible in robotic applications. Robots commonly are deployed in open environments, where they will often come across new types of objects. Recently, Abderrahmane et al. (2018, 2019) proposed a tactile recognition framework, which can recognize both known as well as novel objects. Nevertheless, this framework still did not explicitly consider the nature of open-set. In particular, the set of novel classes that can be recognized must be known in advance in the framework. Moreover, it relied on the hypothesis that attributes learned from the training seen-classes are shared by the testing unseen-classes. Obviously, they are potential drawbacks in practice applications. Consequently, existing methods are not suitable for open-set tactile object recognition.

### 2.2. Open-set recognition

Open-set tactile recognition faces the core challenge is how to not only correctly classify samples from the known classes but also effectively detect and flag unknown examples as the novel. Traditional closed-set classification models may not work in open-set problems because they often predict high confidence for inputs that are significantly different from the training classes (Wang et al., 2022). To tackle this challenge, a variety of related methods have been proposed in the literature. An intuitive method is to use closed-set classifier to solve open-set recognition by setting rejection threshold, such as 1-vs-set SVM (Scheirer et al., 2012), SROSR (Zhang and Patel, 2016), NNO (Bendale and Boult, 2015), DOC (Shu et al., 2017), and CROSR (Dhamija et al., 2018). Exploring this idea, Scheirer et al. (2012) proposed 1-vs-Set model based on SVM to detect unknown samples by adding an extra hyper-line. Bendale and Boult (2015) extended Nearest Class Mean (NCM) classifier to open-set conditions, establishing a Nearest Non-Outlier (NNO) algorithm. Recently, Bendale and Boult (2016) proposed to use the Openmax layer to replace the Softmax layer in deep neural networks. This method redistributes the probability distribution of Softmax to obtain the class probability of unknown samples.

As most of these models ignore constructing reasonable feature distribution for different classes, the learned features generally have excessive intra-class variance (Han et al., 2017). The inter-class distance can even be smaller than the intra-class distance in the learned feature space. As a consequence, it is hard to select an appropriate threshold that well separates known from unknowns. Moreover, feature distribution of training samples is not explicitly considered in their learning objectives, which will limit the performance of the model to detect unknown samples.

Another technical route is to collect or synthesize examples of extra classes for representing unknowns. Along this line, G-OpenMax (Ge et al., 2017) proposed to train a generator for synthesizing examples that represent all unknown classes for model training. Neal et al. (2018) developed counterfactual image generation, which aimed to generate extra class image samples that cannot be classified into any known class. Since the complex collection process and operation constraints, it is difficult to acquire large amounts of tactile data for unknown. Therefore, it is unfeasible to learn an effective model with limited training data for generating sufficient samples to represent unknowns.

## 3. Problem formulation

In this work, we aim to realize robotic tactile object recognition in open-set scenarios. The goal is to endow robots with an effective mechanism to detect samples from unknown classes that may be encountered during testing, which are not available to be seen in training. To accomplish this goal, the tactile open-set recognition model is able to (i) correctly classify known tactile inputs (i.e., classes from the training set) and (ii) effectively detect unknown tactile classes (i.e., classes not exposed in the training set).

Let us formalize the problem described above. Given a tactile training dataset $D_{tr}={\left\{\left(t_{i},y_{i}\right)\right\}}_{i=1}^{M}$, where $t_{i}\in R^{d}$ denotes a training tactile sample, *y*_*i*_∈*Y* = {1, 2, …., *K*} is the corresponding class label and *M* denotes the number of training samples. The testing dataset $D_{te}={\left\{\left(t_{j},y_{j}\right)\right\}}_{i=1}^{N}$ where $t_{j}\in R^{d}$, $y_{j}\in Y^{\prime }=\left\{1,2,....,K,K+1,....,k^{\prime }\right\}$ (*k*′>*K*) and *N* is the number of testing samples. Here, {*k*+1, …., *k*′} denotes the set of unknown categories, which is referred to as novelty and uniformly denoted as *Y*_*K*+1_ in this paper. Therefore, $Y^{\prime }=Y\cup Y_{K+1}$ and *Y*∩*Y*_*K*+1_ = ∅. Our task is that the tactile recognition system need to determine whether a tactile observation $t_{j}\in Y^{\prime }$ is from the known classes *Y* or the unknown classes *Y*_*K*+1_. If *t*_*j*_ is from *Y*, the classifier should predict a class label ŷ∈*Y*, otherwise it can be judged as the novel class *Y*_*K*+1_.

The primary challenge of solving this problem is how to enable the model to classify tactile examples of seen classes into their respective classes and meantime detect tactile data of unseen classes. Traditional classifiers predict the class of the input instance with the highest Softmax probability. Since the model is impossible to know in advance unknown classes that may be encountered in practice, it tends to predict the lowest probability on the unknown classes. As a consequence, directly using closed-set classifiers for open-set recognition would classify unknown instances into known categories with improperly high confidence, yielding poor performance in open-set recognition. What is more, it is hard to collect sufficient tactile data in practice. These factors make the existing open-set methods unsuitable for tactile recognition. Therefore, it needs to be investigated carefully.

As discussed above, the open-set tactile recognition is a non-trivial task due to the following two major challenges:

1. Similar to other open-set recognition problems, open-set tactile recognition also faces the core challenge is how to not only correctly classify samples from the known classes but also effectively detect and flag unknown examples as the novel.
2. Different from other open-set visual recognition tasks, collecting sufficient tactile data is difficult for training due to the complex collection process and constraints of robot-object physical interactions. This makes it difficult to migrate some existing open-set recognition methods with a complicated network to the tactile open-set recognition task.
3. Moreover, the tactile signals for object recognition are commonly high-dimensional dynamic time-series, which exhibit many challenges. Firstly, it is impossible to directly use high-dimensional signals into the existing machine learning methods without any preprocessing techniques. Additionally, there is the nature of misalignment among different tactile measurements. It makes tactile open-set recognition more difficult.

## 4. The proposed method

In this section, we first expound the framework architecture of the proposed method, and then we elaborate the details of the Gaussian prototype learning model in the method. Finally, we describe the algorithm optimization of the model.

### 4.1. Framework architecture

**Figure 2:**
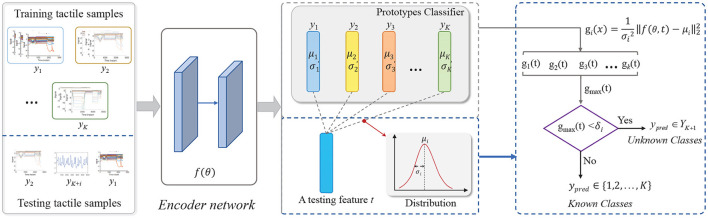
The framework of our proposed method for open-set recognition.

The framework of our proposed model is shown in Figure 2, which can be structurally disentangled into two main modules: *feature extraction module* *f*(θ, *t*) and *Gaussian prototype learning module*. The feature extraction module is used to transform the raw tactile inputs into abstract feature representations, where *t* is a tactile input and θ denotes the parameters of the feature extraction module. Different from the traditional softmax layer for classification on the learned features, we adopt a prototype learning module to learn class prototypes $\mu_{y_{i}}^{l}$ on the extracted features for each class *y*_*i*_∈*Y*, where the superscript *l*∈{1, 2, …, *L*} is the number of prototypes in each category. Finally, we apply these prototypes for classification by template matching. When the extracted feature *f*(θ, *t*) of an input *t* can not match well with all prototypes of all known classes, it can be viewed as unknown. In this model, a feature extraction module and prototype module are jointly learned from data during training, thus forming a unified end-to-end deep framework, which is beneficial to improve the performance of recognition.

Previous experiments demonstrated that when the number of prototypes *l* in each class is large, it can not promote the classification accuracy and on the contrary will degrade the performance of the model. In fact, the deep neural network is very powerful for feature representation. Although the initial feature distribution is complex and scattered, the features of each class can be compacted to fit a single class centroid with some appropriate constraints after transformation. As such, we maintain one prototype for each category in our model. For convenience, $\mu_{y_{i}}^{l}$ is denoted as μ_*y*_*i*__, of which the superscript is omitted in the following description.

### 4.2. Gaussian prototype learning model

Given a tactile input *t*_*j*_, we firstly extract its abstract representation through the feature extraction module *f*(θ, *t*_*j*_), and then search the nearest prototype based on the Euclidean distance between the extracted feature with all prototypes in the feature space. Finally, we assign the class label of this prototype to the tactile input. The process can be described as:


(1) $$\hat{y}=\{\begin{array}{l} \;\arg\overset{K}{\underset{i=1}{\max}}g_{i}(t_{j}),\;if\;g_{i}(t_{j})>\delta \\ Unknown\;Y_{K+1},\;if\;g_{i}(t_{j})\leq \delta \end{array}$$

where *g*_*i*_(*x*) is the class discriminant function that denotes the matching score of tactile sample *t*_*j*_ with class *i*, δ is a rejection threshold.

To train the framework, we introduce the three optimization objectives, which are *discriminative classification loss, feature distribution loss* and *learning to detect unknowns*.

#### 4.2.1. Discriminative classification loss

Intuitively, an ideal class prototype should effectively discriminate and classify samples from different categories. To achieve the goal, we propose a discriminative classification loss. It aims to make the prototype of the corresponding class closer to *f*(θ, *t*_*i*_) while the prototypes of other classes stay away from *f*(θ, *t*_*i*_), ensuring tactile input is correctly classified.

Essentially, the discriminative classification loss is a novel distance-based cross-entropy loss. Similar to traditional cross-entropy loss, it calculates cross-entropy loss with class probabilities obtained from the distances between samples feature and all prototypes. Specifically, given a sample *t*_*i*_ and its class label *y*_*i*_, the probability of belonging to the corresponding prototype can be measured by the distance, and the probabilities are normalized in a similar way of Softmax. With this definition, the loss is defined as:


(2) $$L_{cls}(\theta ,\mu_{i})=-\frac{1}{N}\sum_{i=1}^{N}\sum_{j=1}^{K}\Gamma (\hat{y}=y_{j})\log P_{y_{j}}(\hat{y}|t_{i}).$$

where Γ(·) is symbolic function, and *p*_*y*_*i*__ is class-specific probability, of which the definition can be expressed as:


(3) $$P_{y_{j}}(\hat{y}|t_{i})=\frac{e^{-\frac{1}{T}{\left\|f(\theta ,t_{i})-\mu_{y_{j}}\right\|}_{2}^{2}}}{\sum_{i\in Y}e^{-\frac{1}{T}{\left\|f(\theta ,t_{i})-\mu_{i}\right\|}_{2}^{2}}}$$

where T is a temperature coefficient that is used to control the characteristics of the classifier. We set the value of *T* as the variance σ^2^ in the feature space, in order to normalize the representation space and increase the stability of the system. All classes prototypes μ_*i*_ with *i*∈*Y* and the variance σ^2^ are updated in an online manner.

By minimizing *L*_*cls*_(θ, μ_*i*_), the loss aims to encourage separating the samples from different categories in learned feature space. In particular, this objective is to decrease the distance between samples of the same category and the corresponding prototype, and increase the distance between the sample and all other incorrect prototypes. Since the objective considers all prototypes in each updating step, it can better guarantee the convergence of training.

#### 4.2.2. Feature distribution loss

For open-set recognition, the learned features need not only to be separable in different classes but also be compact in the same class. However, the above classification loss only makes the features of different categories separable. As a result, a feature *t*_*i*_ is far away from the corresponding category centroid μ_*y*_*i*__, but it still is correctly classified if it is relatively closer to μ_*y*_*i*__ than to the feature centroids of the other classes. To tackle this issue, we further introduce a feature distribution loss to learn discriminative and compact representation, making it more applicable for our task.

The feature distribution loss is essentially the maximum likelihood regularization term on the assumption of Gaussian distribution. Specifically, we assume that the extracted feature on the training set conforms the Gaussian mixture distribution, viewing class prototype μ_*y*_*i*__ as the mean of a Gaussian component, which can be expressed as:


(4) $$\begin{array}{l} p\left(t_{i}\right)=\overset{k}{\underset{i=1}{\sum }}N\left(f\left(\theta ,t_{i}\right),\mu_{y_{i}},\sigma_{y_{i}}\right)p\left(y_{i}\right). \end{array}$$

where σ_*y*_*i*__ is covariance of class *y*_*i*_ in the feature space, and *p*(*y*_*i*_) is the prior probability of class *y*_*i*_. For the convenience of calculation, the likelihood regularization term is defined as the negative log-likelihood. By reasonably setting constant prior probabilities *p*(*y*_*i*_), the likelihood regularization term *L*_*lkd*_ is simplified to Equation (5).


(5) $$\begin{array}{l} L_{lkd}\left(\theta ,\mu_{y_{i}}\right)=-\overset{k}{\underset{i=1}{\sum }}\log N\left(f\left(\theta ,t_{i}\right),\mu_{y_{i}},\sigma_{y_{i}}\right). \end{array}$$

The objective of the regularization term aims to maximize the log-likelihood of sample *t*_*i*_ for its corresponding class.

By minimizing *L*_*lkd*_, the model can effectively reduce the within-class variance and constrain the feature distribution of known classes, so it can reserve more feature space for unknown classes and improve the performance of the proposed method for detecting unknowns.

#### 4.2.3. Learning to detect unknowns

The threshold-based rejection is frequently used in open-set recognition tasks. Most of the existing methods directly adopt the predefined threshold to detect unknowns, which is not suitable in practical applications. In order to make our model effective on the open set tasks, we explicitly consider adopting class-specific rejection criteria. In particular, we use an adaptive strategy by letting the value threshold δ to be proportional to maximal distance Δ_*y*_*i*__ between samples specific class *y*_*i*_ and the corresponding class centroid μ_*y*_*i*__, i.e., δ = αΔ_*y*_*i*__ where α is proportional coefficient. Formally, Equation (1) can be expressed as:


(6) $$\hat{y}=\{\begin{array}{l} t\in class\;\arg\overset{k}{\underset{i=1}{\max}}g_{i}(t),\;if\;g_{i}(t)>\alpha \Delta_{y_{i}}\\ Unknown,\;if\;g_{i}(t)\leq \alpha \Delta_{y_{i}} \end{array},$$

where $g_{i}\left(t\right)=\frac{1}{\sigma_{y_{i}}^{2}}||f\left(\theta ,t\right)-\mu_{y_{i}}|{|}_{2}^{2}$. Instead of adopting the pre-defined threshold, we explicitly learn specific threshold of each category by minimizing the following objective:


(7) $$\begin{array}{l} L_{thr}\left(\theta ,\mu_{y_{i}}\right)=\underset{i\in Y}{\sum }\max\left(0,m\left(\frac{1}{\sigma^{2}}{\left||f\left(t_{i},\theta \right)-\mu_{y_{i}}|\right|}^{2}-\alpha \Delta_{i}\right)\right). \end{array}$$

where *m* = −1 if *i* = *y*_*i*_ and *m* = 1 otherwise.

By minimizing *L*_*thr*_, the model can obtain class-specific rejection thresholds, instead of presetting a global threshold as in prior works. It makes the proposed model effective to detect unknown samples.

### 4.3. Algorithm optimization

With the above-mentioned analysis, the optimization process of our proposed method is structurally divided into two components: *optimization of feature representation* and *optimization of rejection threshold*.

(1) In this optimization of feature representation, the trainable parameters in the proposed method are composed of two parts, i.e., parameters of encoder network for feature transformation *f*(θ, *t*_*i*_) and all classes prototypes μ_*i*_. To this end, we combine discriminative classification loss and feature distribution loss. The formally objective function is expressed as:


(8) $$\begin{array}{l} L\left(\theta ,\mu_{y_{i}}\right)=L_{cls}\left(\theta ,\mu_{y_{i}}\right)+\lambda L_{lkd}\left(\theta ,\mu_{y_{i}}\right) \end{array}$$

where λ≥0 is weighting coefficients, which controls the trade-off of the two loss terms to optimal performance. For the hybrid optimization objective function *L*(θ, μ_*y*_*i*__) in Equation (5), we can directly calculate the gradients of ∂*L/∂f* and ∂*L/∂μ*_*y*_*i*__. According to the error back propagation, we can calculate the gradient of ∂*L/∂θ*. With the gradients of *L* over all parameters, we can jointly optimize both feature extractor and all classes prototypes using a gradient descent (SGD) optimization algorithm in an end-to-end way.

(2) For optimization of rejection threshold, it aims to achieve optimal class-specific thresholds αΔ_*i*_. In this process, we held-out set of samples from the training set to learn the optimal thresholds. Hence, we split the samples into two parts, one part used for learning the feature extractor *f*(θ) and the classes prototypes μ_*y*_*i*__ and the remaining part for learning the value of αΔ_*y*_*i*__.

In summary, the optimization process of our proposed model is elaborated in Algorithm 1.

**Algorithm 1:**
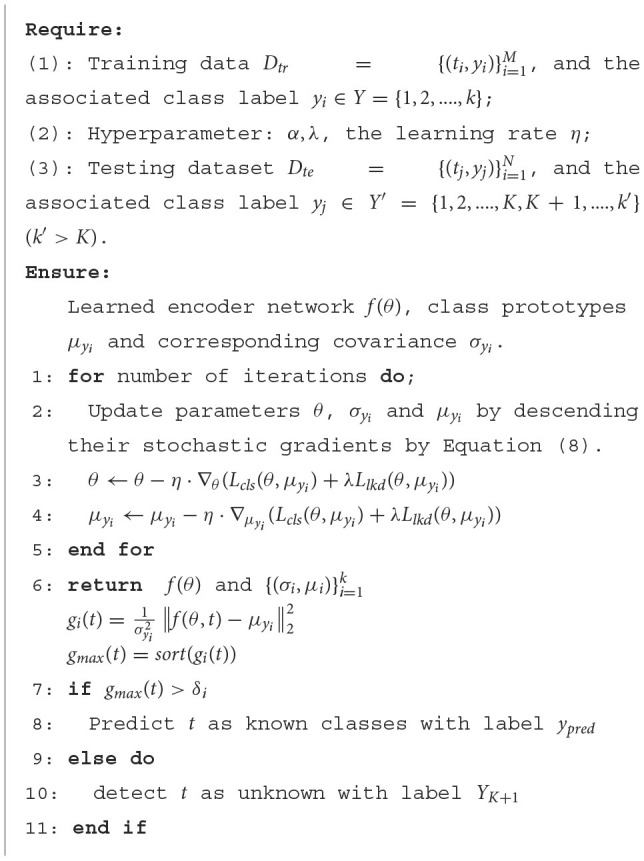
The program flowchart of the proposed method.

## 5. Experiments

In this section, the proposed open-set tactile recognition method is comprehensively evaluated on two publicly available datasets. Firstly, the adopted datasets, evaluation metrics, comparison methods, and implementation details are described. Then experiments results and their analysis are provided. Finally, we further analyze the sensitivity of hyperparameters in the model.

### 5.1. Dataset splits

**Figure 3:**
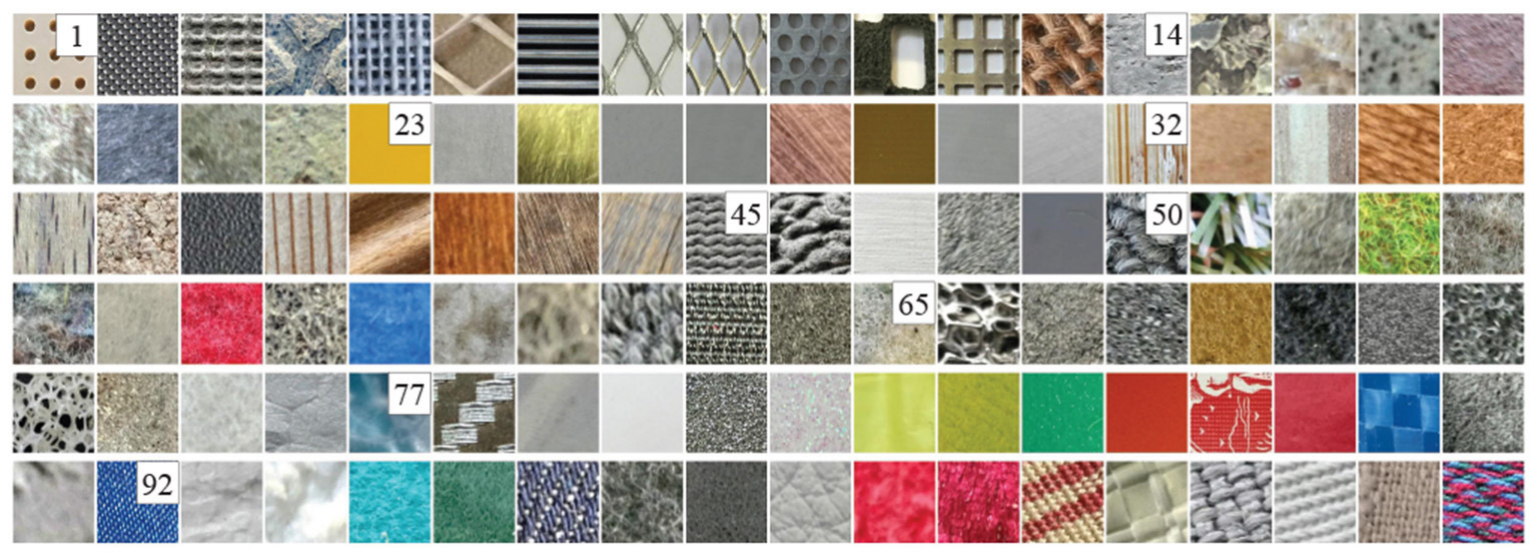
All material images of the data set. The numbers, respectively, denote the beginning of each category. The original images are from Strese et al. (2016). It has been reproduced with permission from IEEE, available at https://zeus.lmt.ei.tum.de/downloads/texture/.

We demonstrate our proposed method on two publicly available data sets, which are Haptic Texture Database (LTM_108) dataset (Strese et al., 2016) and Penn Haptic Adjective Corpus (PHAC-2) (Chu et al., 2015) dataset. They have been used to evaluate a model's ability to recognize objects or textures by tactile modality (Liu and Sun, 2017). In these two data sets, their tactile data, respectively, represent two typical types of tactile information. Different from closed-set recognition, open-set tactile recognition needs a special setup and experiments. The splitting of the dataset is described as follows:

**LTM_108:** The LTM_108 dataset consists of 108 different surface material instances, which are divided into 9 categories based on the material properties. These material images of the dataset are shown in Figure 3. In this dataset, it provides multimodal data for each material instance, namely visual images, tactile acceleration traces and sound signals generated from the surface-tool interaction. The dataset provides a training set and a testing set. They both contain 108 material instances and every instance has ten tactile samples. In this experiment, we only use the tactile acceleration traces as tactile data for object recognition.

Although this dataset has been directly used for some closed-set tasks of tactile recognition, we use this dataset to tackle more challenging the open-set tactile recognition task. To provide a suitable test platform, a new dataset split is proposed based on the original dataset. In particular, we randomly select *K* < 9 categories tactile samples from the train set to train our models and use totally 9 categories of tactile samples from the test set for test evaluation. This setting ensures that the test set appears some material categories that are not in the training set. The Table 1 show a case of the dataset splits when *K* = 6.

**Table 1:** The details of the dataset splits on LTM_108.

**Material category**	**Training samples**	**Testing samples**
Mesh	13 × 10	13 × 10
Stones	9 × 10	9 × 10
Glossy	9 × 10	9 × 10
Wood	13 × 10	13 × 10
Rubbers	5 × 10	5 × 10
Fibers	15 × 10	15 × 10
Foams	-	12 × 10
Foils and paper	-	15 × 10
Textile and fabrics	-	17 × 10
Total	64 × 10	108 × 10

**Figure 4:**
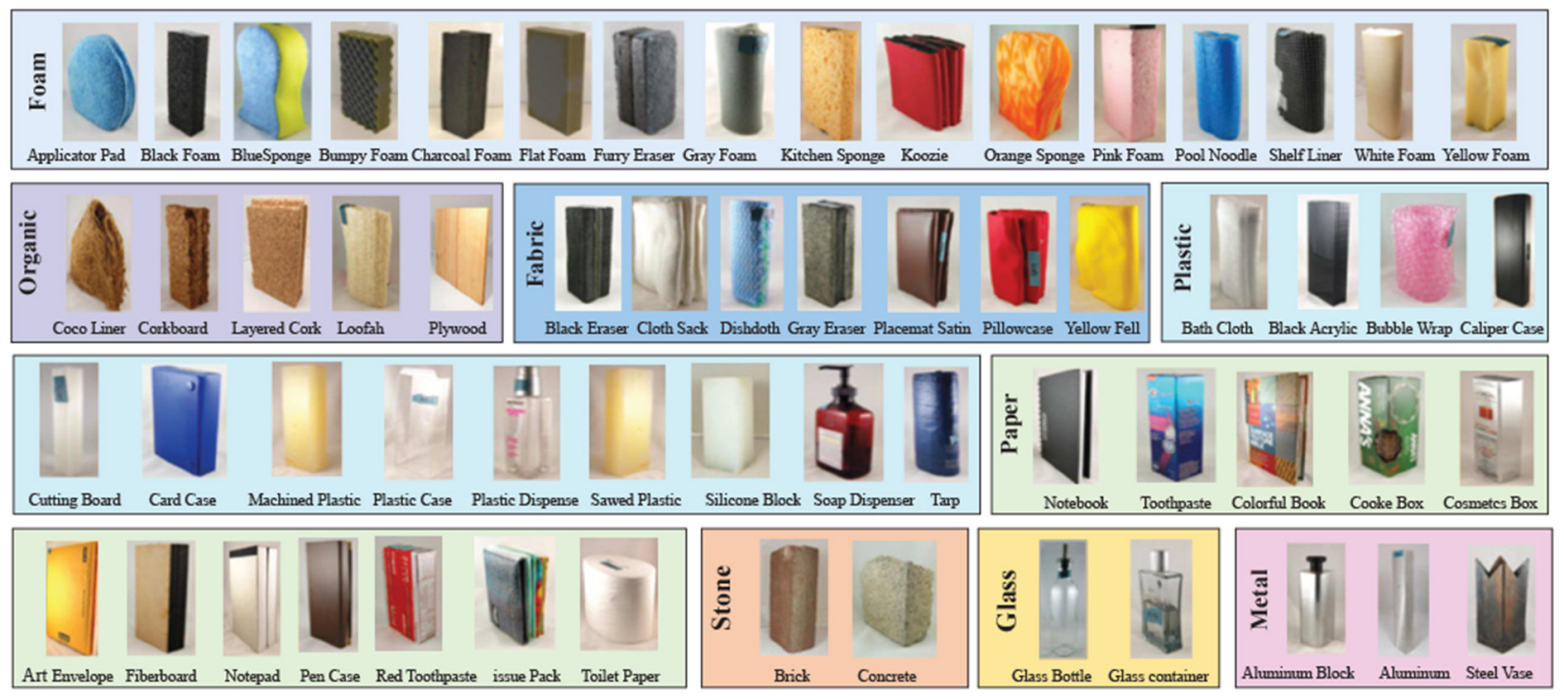
The PHAC-2 contains 60 objects, which are organized by their primary material. The original images are from Chu et al. (2015). It has been reproduced with permission from Elsevier, available at https://hi.is.mpg.de/research_projects/learning-haptic-adjectives-from-tactile-data.

**PHAC-2:** There are 60 objects in the PHAC-2 dataset. The visual images of the dataset are shown in Figure 4. According to the physical properties, these objects are divided into eight categories. In this data set, each object contains tactile signals and visual images. The tactile signals are collected by two SynTouch BioTacs tactile sensors, which are installed to the grippers of a PR2 robot. In order to mimic the process of humans exploring the tactile properties of objects, the robot used four exploratory procedures to acquire five types of tactile data. According to the specified procedures, ten trials are performed on each object, resulting in totally 600 tactile samples. Although the joint data on gripper during exploratory movements are available, we focused on the tactile signals for classification in this experiment. In particular, each tactile sample consists of five components *P*_*DC*_, *P*_*AC*_, *T*_*DC*_, *T*_*AC*_ and *E*_19_.

Similar to the above dataset setting, the PHAC-2 dataset also needs to be reorganized and split. Firstly, we randomly select an object instance from each material category as test objects and remain other 52 object instances. Then, we randomly select *K* < 8 categories from eight categories from the remaining object instances. When *K* = 5, the details of the dataset splits are shown in Table 2. Please note that according to the above setting, not only does the test set contains some categories that are not in the training set, but also the training set and the testing set do not share the same object instance even from the same category. Different from instance-level recognition, this experiment can be referred to as categorization-level open-set recognition. To this need, we need the proposed model to have generalization and robustness for unseen object instances.

**Table 2:** The details of the dataset splits on the PHAC-2.

**Material category**	**Original samples**	**Training samples**	**Testing samples**
Foam	16 × 10	15 × 10	1 × 10
Organic	5 × 10	4 × 10	1 × 10
Fabric	7 × 10	6 × 10	1 × 10
Plastic	13 × 10	12 × 10	1 × 10
Paper	12 × 10	11 × 10	1 × 10
Stone	2 × 10	-	1 × 10
Glass	2 × 10	-	1 × 10
Metal	3 × 10	-	1 × 10
Total	60 × 10	48 × 10	8 × 10

### 5.2. Data preprocessing and network architecture

Considering the difference between the two types of tactile signals, we adopt two different feature extraction methods and network architectures for classification. The specific details are as follows.

**LTM_108:** In the LTM_108 dataset, the recorded tactile signals are three-axis acceleration traces. Firstly, the three-axis acceleration traces are conversed to a one-dimensional signal by the DFT321 algorithm (Kuchenbecker et al., 2010). Considering the effectiveness of short-time Fourier transform (STFT) extracting features of time-series signals, we adopt STFT to convert a one-dimensional DFT321 signal into a spectrogram. These spectrograms are in the log domain, where the length of a frame length is 500 and the increment of frame and frame is 250. By the predefined configuration settings mentioned above, there are 100 spectrogram samples of size 50 x 250 extracted from each tactile acceleration trace.

As convolutional neural network (CNN) has proven to be effective in visual classification, which has achieved good performance on many tasks. Moreover, some CNN models pre-trained on ImageNet (Deng et al., 2009) have shown generalization and discrimination. In this experiment, we use the pre-trained Resnet18 (He et al., 2016) model on ImageNet as the network backbone of the proposed method.

**PHAC-2:** As in Abderrahmane et al. (2019), we firstly normalize the five components (*P*_*DC*_, *P*_*AC*_, *T*_*DC*_, *T*_*AC*_, *E*_19_) in each signal sample, respectively. As the sample rate of *P*_*AC*_ is higher than other components of a tactile sample, we downsample it to match the other signals' sample rate of 100 Hz. For some exploratory movements, the length of tactile signals varies considerably from objects. In order to resolve the length difference of signal, we downsample the signal of each exploratory movement to a fixed length of 150. Principal Component Analysis is used independently on the *E*_19_ data from each exploratory movement to capture the four most principal components across all objects. Thus, we obtain 64 tactile signals for each object in each trial.

Recently, Ji et al. (2015) has demonstrated the effectiveness of CNN on temporal signals with limited amounts. In this experiment, we adopt Convolutional neural networks (CNN) to perform tactile object recognition. The specific network structure is the same as the Haptic CNN model in Gao et al. (2016). Every tactile sample per object has 64 tactile signals. We concatenate the 64 features along the channel axis, which is used as the input of our model.

### 5.3. Evaluation metric

In this experiment, we use the three metrics to evaluate the classification performance, including *Accuracy* and *F-measure* and *AUC*.

- Accuracy: As a common metric method to evaluate classifiers on a closed set task, recognition accuracy *Acc* is defined as:

(9) $$\begin{array}{l} Accuracy=\frac{\text{TP + TN}}{\text{TN + TP + FP + FN}} \end{array}$$

where TP, TN, FN, and FP, respectively, denote true positive, true negative false negative, and false positive. The sum of the three quantities is equal to the total number of samples.
• F-measure: F-measure is commonly evaluation metric, which is defined as a harmonic mean of Precision *P* and Recall *R*:

(10) $$\begin{array}{l} F-measure=2\times \frac{P\times R}{P\times R}=\frac{2TP}{2TP+FP+FN} \end{array}$$

As suggested in Bendale and Boult (2016) and Geng et al. (2020), we use macro-averaged F1-score. It is denoted as macro-F1.
• AUC: It denotes area under the ROC curve (AUC), which measures the performance of detecting unknown between known and unknown data.

### 5.4. Comparison methods

To validate the advantages of our proposed method, several classical methods were also implemented for comparison. A brief description of the methods is as follows:

- Softmax: It used the highest probability from the softmax layer of networks as the confidence score for classification.
- τ-Softmax (Hendrycks and Gimpel, 2016): It aims to use a global threshold on the softmax probability to determine whether an input sample belongs to an unknown class. We refer to this method as τ-Softmax.
- τ-Center (Wen et al., 2016): It can be combined with cross-entropy loss to encourage the training data to form better-behaved class structures, which may be easier to model and facilitate greater distinction of open-set inputs. To this end, we also use it to detect unknown classes by a predefined threshold, which is denoted as τ-Center loss.
- OpenMax (Bendale and Boult, 2016): It proposed replacing the softmax layer with OpenMax, which calibrates the confidence score with Weibull distribution. It proposed an inference method for detecting novel classes.

We note that some advanced methods, such as Yoshihashi et al. (2019) and Sun et al. (2020), have also been proposed to deal with open-set visual recognition. However, we do not take them for comparison, because the networks of these methods are too complex to work on the limited training data of tactile tasks.

### 5.5. Implementation details

For open-set recognition, the ratio of seen and unseen is an important factor, which quantifies the openness of the problem. As in Zhou et al. (2021), the openness is defined as:


(11) $$\begin{array}{l} openness=1-\sqrt{\frac{N_{train}}{N_{test}}} \end{array}$$

where *N*_*train*_ and *N*_*test*_, respectively, denote the number of categories in training set and testing set. As we described in the preliminaries, *N*_*train*_ = *K*.

In this experiment, we empirically set the likelihood regularization parameter λ to 0.01 in experiments. For the margin parameter α, the optimization of the objective function becomes more difficult as the value increases. Therefore, α needs to be smaller when the number of classes gets more. In our experiments, we empirically set α to 0.4 and 0.3 for LTM_108 and PHAC-2, respectively.

### 5.6. Experimental results and analysis

Experimental results on the LTM_108 and PHAC-2 datasets are reported in this subsection. In this experiment, we randomly select 6 categories as known classes for LTM_108. Considering the instance imbalance of categories in PHAC-2, the first five categories are used as known classes. Ten trials are performed on each experiment, and the averaged results are used as final metric scores. In this setting, the corresponding experimental results on different methods are shown in Table 3.

**Table 3:** Experimental results of different method.

**Model**	**LTM_108**			**PHAC-2**		
	**Accuracy**	**Macro-F1**	**AUC**	**Accuracy**	**Macro-F1**	**AUC**
Softmax	59.1%	0.491	0.878	62.5%	0.518	0.871
τ-Softmax (Hendrycks and Gimpel, 2016)	61.7%	0.567	0.970	70.01%	0.625	0.975
τ-Center (Wen et al., 2016)	64.9%	0.613	0.975	71.3%	0.634	0.977
OpenMax (Bendale and Boult, 2016)	62.5%	0.574	0.978	58.7%	0.536	0.928
Proposed method	70.76%	0.669	0.986	75.5%	0.703	0.986

From Table 3, it can be seen that our proposed method achieves the highest Acc, macro-F1, and AUC on the two datasets. It indicates that the proposed model outperforms the compared methods, which also demonstrates that the proposed method is able to effectively improve the ability to detect unknowns while ensuring the accuracy of the known classification simultaneously.

As mentioned above, the open-set recognition on PHAC-2 is more challenging, as its test set and training set does not share the same object instance. Besides, we do not perform any data augmentation or employ some specific and complex networks in these experiments. Even so, our proposed method still achieves optimal performance. This further verifies the effectiveness of the proposed method.

Additionally, it is clear that τ-Center exhibits a better performance among all these compared methods because it explicitly encourages stronger compactness of feature, which is beneficial for open-set recognition. However, it mainly aims at improving the softmax loss and feature distribution is not explicitly modeled. Therefore, it can not achieve optimal performances dealing with the tactile OSR problem. Since integrating the advantages from both classification discrimination with Gaussian Prototype Learning and likelihood estimation of feature distribution, our proposed method performs better in open-set conditions. It highlights the importance of considering the likelihood of feature distribution in the tactile OSR problem.

In particular, we can observe that as a state-of-art open-set recognition method, Openmax shows low performance, especially on the PHAC-2 dataset. It is mainly due to low recall on known classes with a few training instances since test instances from smaller classes are usually projected farther from the mean activation vector of the corresponding class. This demonstrates that Openmax may be also hardly infer the class probability of unknown inputs by the probability distribution of Softmax. Moreover, our experiments indicate that merely thresholding the output probabilities of softmax helps, but is still relatively weak for open set recognition.

### 5.7. Effectiveness of different openness

**Figure 5:**
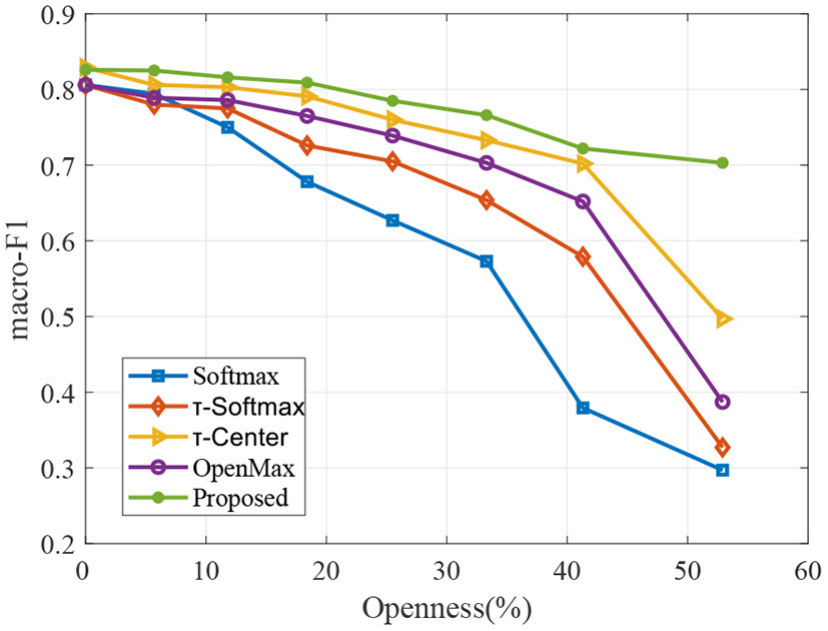
Macro F1 against varying openness with different methods on the LTM_108 dataset.

**Figure 6:**
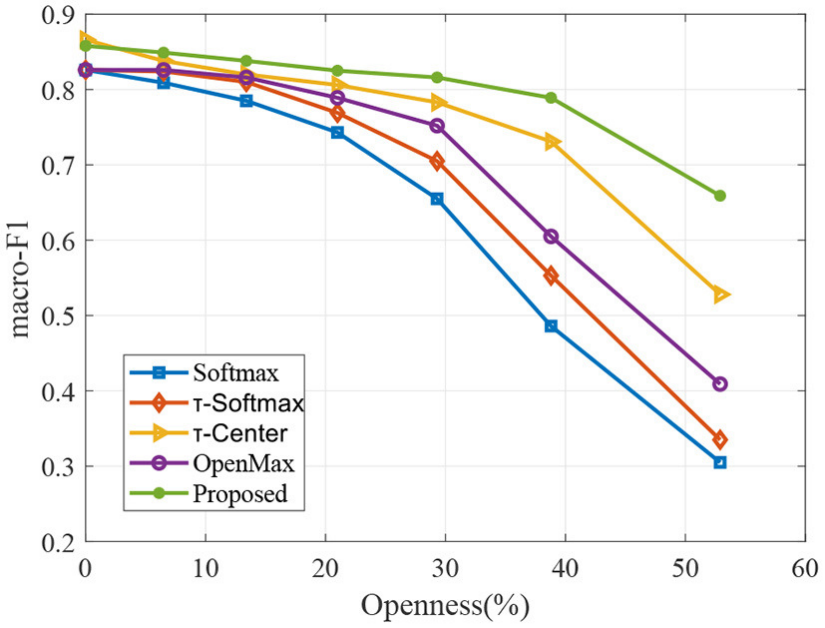
Macro F1 against varying openness with different methods on the PHAC-2 dataset.

To valid the robustness of our proposed model to different openness, we evaluate performance over multiple openness values in the experiments. In particular, we vary the openness of Equation (11) by varying the number of classes in the training sample, while the number of test classes remains the same. We evaluate the performance by macro F1-scores. The corresponding results are shown in Figures 5, 6.

As to be expected, when more known classes are available during training, the performances of classifiers are better for all methods in Figures 5, 6. We can observe that the proposed approach remains relatively stable over a wide range of openness, which produces better results compared to other methods.

### 5.8. Parameter sensitivity analysis

In the proposed model, α and λ are important parameters, and their values affect on the model's performance. To obtain optimal values for these parameters, we conduct extensive experiments to perform grid search for α and λ within the set {0.1, 0.2, 0.3, 0.4, 0.5, 0.6, 0.7, 0.8, 0.9, 1.0} and {0.0001, 0.001, 0.01, 0.1, 1, 10, 100}. The experimental results show that the model can achieve optimal performance when α = 0.4 and λ = 0.01 on the LTM_108 dataset. For the PHAC-2 dataset, the model shows the best performance where α = 0.3 and λ = 0.01. For the convenience of explanation, the sensitivity analysis of these two parameters is divided into two parts for illustration.

**Figure 7:**
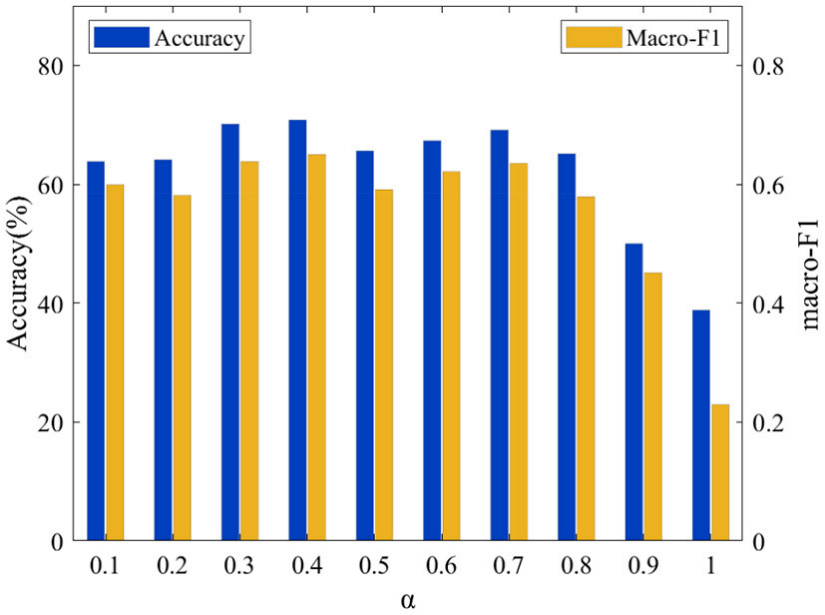
Acc and macro F1 for different α on the LTM_108 dataset.

**Figure 8:**
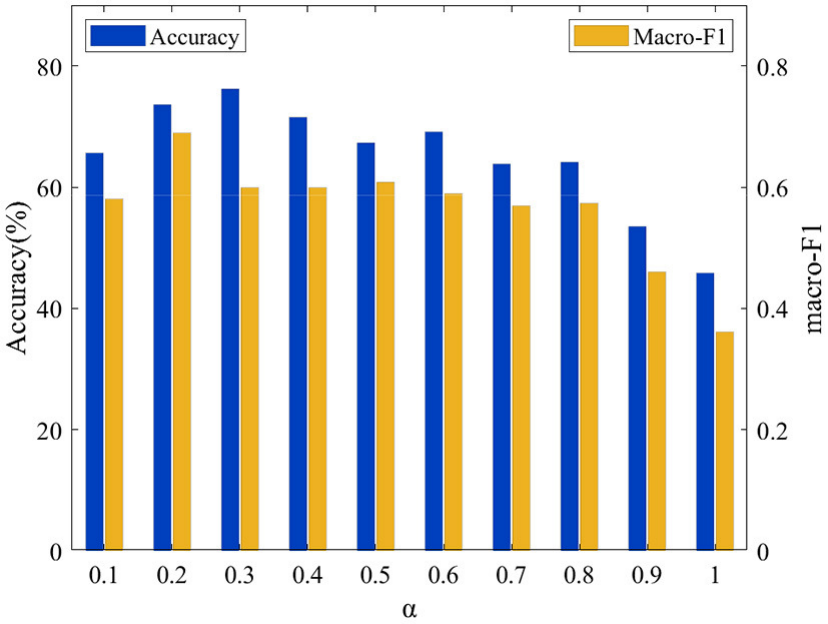
Acc and macro F1 for different α on the PHAC-2 dataset.

To analyze the effect of these parameters α on the proposed model's performance, we set the value of λ to 0.01 and perform grid search of the parameter α within the set {0.1, 0.2, 0.3, 0.4, 0.5, 0.6, 0.7, 0.8, 0.9, 1.0}. The relationships between Accuracy and the macro-F1 and of the value of α are shown on the two datasets in Figures 7, 8, respectively. It can be observed that the performance of the model is very sensitive to the value of the parameter α, and the model performs well when α∈[0.1, 0.8] on both datasets.

**Figure 9:**
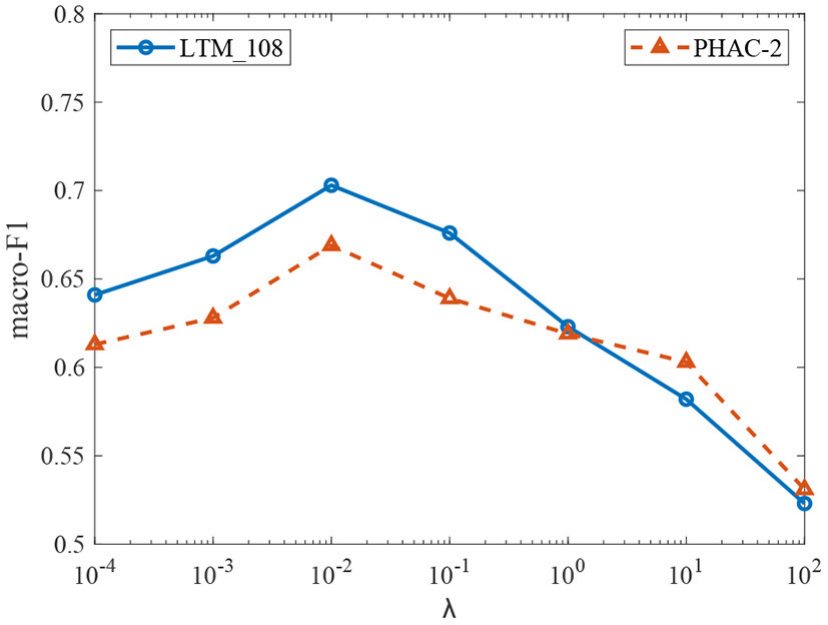
The performance of proposed model in terms of λ.

Then, we conduct experiments to study the effect of the parameter λ on the performance of the model. Fixing parameters α = 0.4 on the LTM_108 dataset and α = 0.3 on the PHAC-2 dataset, we tune the parameter λ within the set {0.0001, 0.001, 0.01, 0.1, 1, 10, 100} and the corresponding experimental results are given in Figure 9. It can be observed that our model achieves good performances in the range of λ∈(0, 1]. When λ>1, the model's performance on the contrary degrades. This is mainly because the likelihood regularization starts to play a role when the training accuracy is close to saturation, and a strong regularization weakens the discrimination effect of the model. Hence, there is a need to find the optimal balance of the two terms in the optimization process.

## 6. Conclusion

In this work, we specifically address tactile object recognition in open-set scenarios, which aims to enable robots to exploit tactile explorations in unstructured environments. To this end, we proposed a novel Gaussian prototype learning model, which incorporates classification and novel class detection into a unified framework. In particular, a likelihood regularization term is introduced to explicitly consider the feature distribution of tactile data. In addition, we further develop an adaptive classification margin to improve the performance of the model. Experimental results validate the effectiveness of the proposed method, which has the potential to improve the performance of open-set tactile perception. We believe that it makes the first step to formulate lifelong tactile recognition in the real world. In the future, we will explore the generalization of the proposed method to realize active continual learning in the open world.

## Data availability statement

The raw data supporting the conclusions of this article will be made available by the authors, without undue reservation.

## Author contributions

WZ and HL proposed the basic idea of this method and completed theoretical modeling. DG performed the experiments analysis and revised the manuscript. FS provided overall supervision of this research. All authors contributed to the article and approved the submitted version.
